# Status of blood transfusion services in Iran

**DOI:** 10.4103/0973-6247.39505

**Published:** 2008-01

**Authors:** A. Gharehbaghian, H. Abolghasemi, M. Tabrizi Namini

**Affiliations:** *Iranian Blood Transfusion Organization-Research Center*

Iranian Blood Transfusion Organization (IBTO) is the only nationally accredited organization in Iran that performs blood transfusion procedures ranging from blood donor recruitment as well as blood distribution. IBTO was established in May 1974. This government-based organization provides its services free of charge. Before its establishment, blood services were provided through hospital-based systems. IBTO is managed by the Supreme Council, which consists of five experts in hematology and related fields appointed by the Minister of Health. The Managing Director of IBTO, elected by the Supreme Council, ensures proper implementation of the decisions adopted. The financial resources of IBTO are covered by a government-approved budget.[1]

The mission of IBTO is to provide and ensure a safe and adequate blood supply in Iran. IBTO fulfils its goals through 30 regional blood centers, which are located in 30 different provinces with more than 200 blood donation sites throughout the country to meet the demands of the Iranian community for blood. It also aims at promoting transfusion medicine in Iran.

## Blood Collection

**Figure 1:**
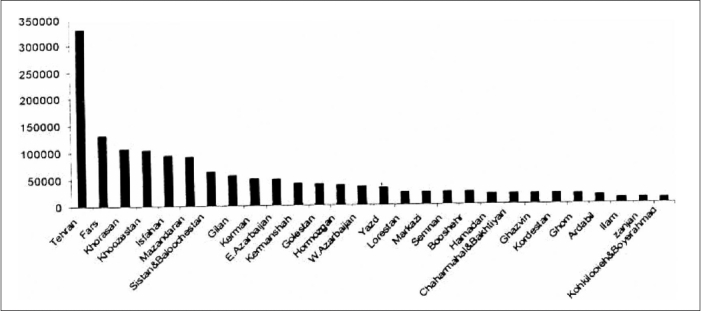
Number of blood donations in each province in the period of March 2003-2004 in Iran.

According to the most recent estimations, there are 23 blood donors per 1000 population. Number of blood donations in each Iranian province in March 2003-2004 is shown in Figure 1.[1] Table 1 shows the number of blood donations per 1000 population in some other countries.[2,4,5,6]

**Table 1:** Number of blood donations per 1000 population

Country	Number of donations/1000 in:			
	
	1993-1995	1997	1999	2001/2002
Argentina	23	21	22	21
Bolivia	5	5	3	29
Brazil	16	15	10	17
Chile	9	11	15	14
Colombia	15	16	9	10
Cor	9	9	24	13
Ecuadore	9	6	8	6
El-salvador	5	0	11	11
Guatemala	5	5	3	6
Honduras	11	10	7	6
Mexico	10	15	11	10
Nigeria	7	8	9	9
Panama	35	8	15	14
Paraguay	10	35	9	8
Peru		11	12	6
Uruguay			35	30
Venezuela			13	15

**Figure 2:**
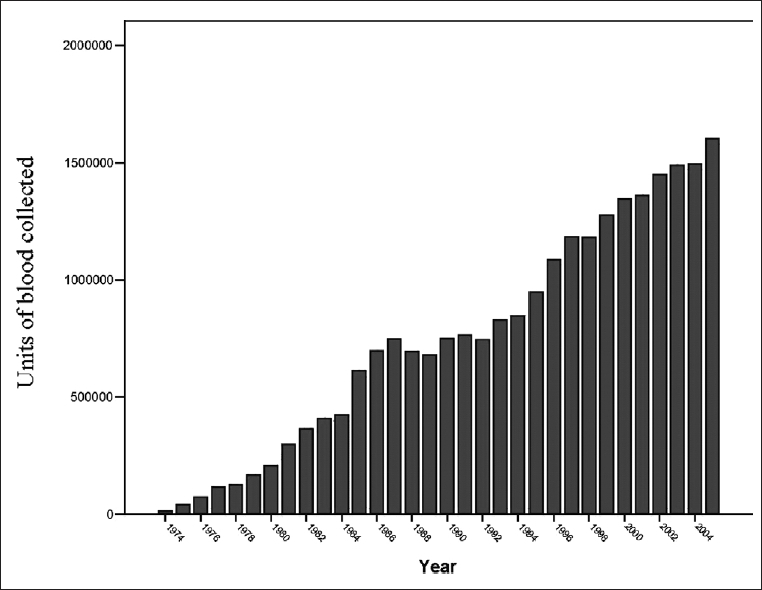
Blood units collected in 1974-2005.

In 2005, the IBTO produced 1,600,000 units of blood for transfusion purposes. Figure 2 shows clearly the proportion of blood for clinical use, as collected from volunteer donors, has increased since 1974. Approximately 96% of blood donations in Iran are collected from voluntary non-remunerated blood donors and the rest (4%) is donated as family replacement donation. This happens in some parts of Iran with socio-economic difficulties and this type of blood collection should be ruled out by the end of this Iranian calendar year (1385 corresponding to 21 March, 2007). At present time (2008), 100% of blood donations in Iran are collected from voluntary non-remunerated blood donors with no replacement whatsoever.

The percentage of blood deferral (13%) is very high in Iran. Only 43% out of the all the blood donors in Iran are repeat donors, while in EU this figure is >90%.[1]

The most effective strategy of IBTO for a successful mission accomplishment for ensuring adequate and safe blood supply was to raise the awareness and motivation of different target populations by publishing books, pamphlets and brochures, holding workshops and making media publicity efforts. These attempts made the proportion of regular blood donors to the total number of blood donors 43%; it is 46% worldwide.

## Units of Blood Collected

**Figure 3:**
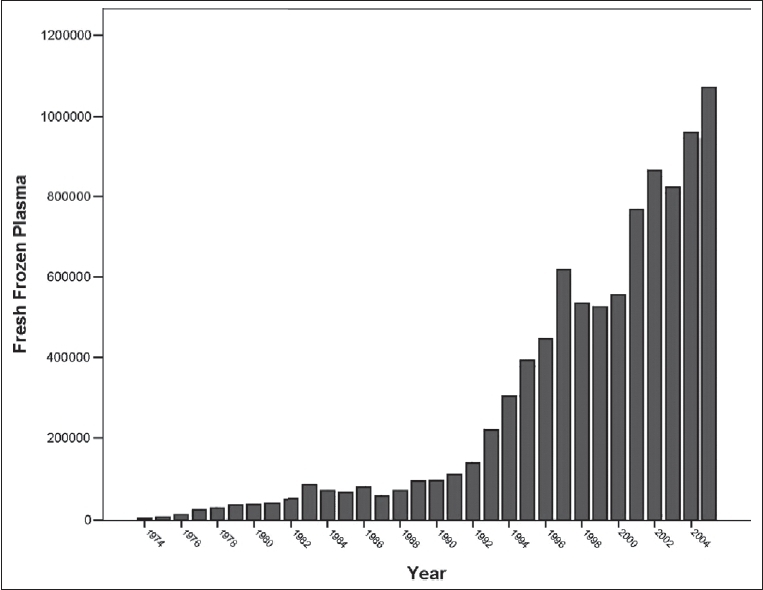
FFP units produced in IBTO during 1974-2005.

**Figure 4:**
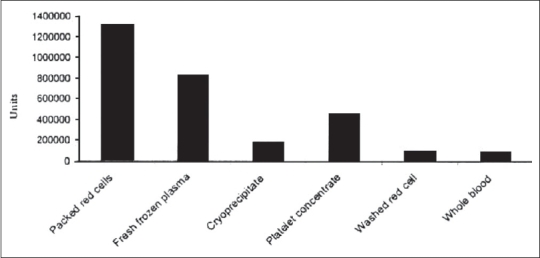
Number of blood components prepared during March 2003-2004 in Iran.

**Figure 5:**
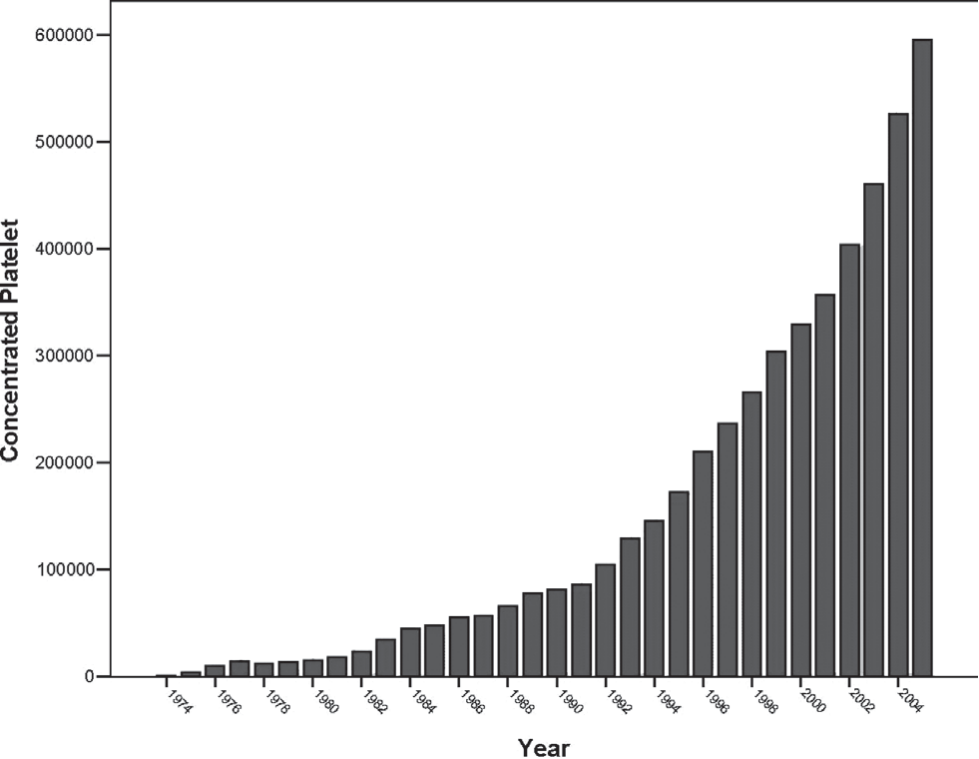
Growth rate of platelet concentrate preparation.

**Figure 6:**
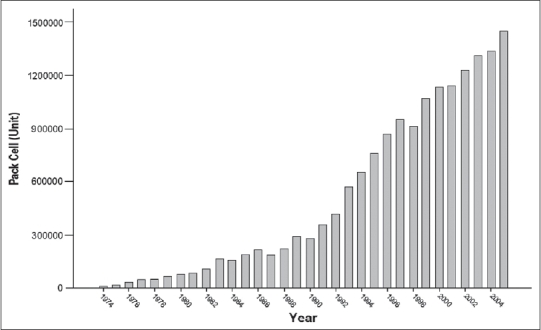
Growth rate of RBC preparation.

The whole blood collection peaked during the Iran-Iraq war (1980-1988), but now it has substantially decreased. Fresh frozen plasma, an important component of blood, which is the source of anti-hemophilic factors and other trace components of plasma, is increasing in terms of its production rate [Figure 3]. The recent strategy of IBTO is to collect platelet through apheresis to promote blood services. The number of blood components prepared during March 2003-2004 in Iran is shown in Figure 4.[1] The growth rates of platelet concentrates and RBCs are shown in Figures 5 and 6.

## Blood Transfusion

One of the most important and successful programs of IBTO is the training of specialists on the use of different cellular and plasma components instead of whole blood. Considering the importance of the production of blood components from whole blood donation, training programs have been held to ensure reduction of blood demands followed by lower rate of whole blood use and higher use rate of packed red blood cells [Figure 6]. On the other hand, given the increase of the number of universities of medical sciences across Iran, the increasing number of subspecialists and the establishment of various specialty and subspecialty wards for major surgeries, and transplantation centers and hematology sectors for better recognition of needs of blood recipients, the production of cellular and plasma components in IBTO has become more specific. It is now primarily based on the special needs of patients thereby resulting in a higher production rate of the components like platelet concentrates. In Iran, a whole blood use analysis shows that the percentage of blood transfused as whole blood is now <10%.[1]

## Blood Donor Recruitment

Selection of low risk donors in pre-donation consultation and interview sessions based on behavioral, medical and demographic factors has improved the transfusion safety even before specific lab screening tests have been conducted; it has also resulted in a lower rate of seropositivity and seroconversion among volunteer blood donors compared with general population. Standardization of criteria for donor eligibility and exclusion, and donor screening procedures are all priorities. Sixty percent of blood donors in Iran are regular, 28% have previous experience with blood donation and 12% are first-time donors. The rates of potential blood donors being deferred are 25, 28 and 28% in 1382, 1383 and 1384 (according to Iranian calendar) respectively. Table 2 shows the number of potential blood donors and those deferred as well as the exact number of blood components prepared.

**Table 2:** Blood collection, voluntary blood donations and blood components in IBTO

Washed blood cells	Platelet	cpp	Cryo	FFP	Packed cells	Whole blood	Voluntary donation (%)	Blood collection	year
104993	403738	187310	176508	864490	1236237	135839	96	1448149	1381 (2002)
102540	460515	180725	184533	823100	1315420	99301	96	1489935	1382 (2003)
94235	514979	201483	196185	931939	1308404	67366	96	1452039	1383 (2004)
101130	594873	233128	236823	1069044	1458374	59559	97	1603149	1384 (2005)
91574	666931	213798	202274	1182434	1531591	49336	99	1667412	1385 (2006)

**Figure 7:**
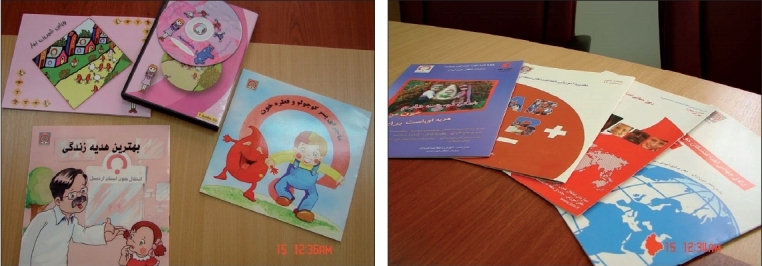
Motivational material.

## Donor Testing

Considerable improvements have been made to detect HBV and HCV, two transfusion transmittable viruses bearing significant morbidity and mortality. The screening tests are conducted to detect HBS Ag, anti HCV, anti HIV-1/2 and serologic tests for syphilis (STS) to provide safety for blood transfusion. Moreover, confirmatory tests are conducted for initially positive tests. All blood units positive for confirmatory tests are not considered.

Despite these improvements, we are seeking ways to obtain even more safe blood and blood components through applying run-control programs, using external controls, operating self-exclusion more seriously and applying viral inactivation techniques. It is a fact that the recruitment of regular voluntary blood donors is the best way to improve blood adequacy and safety.

The index of blood transfusion transmitted infections has been decreasing steadily over time in most countries including Iran in tandem with the increase in screening coverage. There is, however, no estimation of residual risk for transfusion transmitted viral diseases in Iran. At present, the prevalence rates of anti-HCV, anti-HIV and HBsAg among blood donors are 0.065%, 0.014% and 0.8%, respectively.

## Distribution

IBTO in 1384 (corresponding to 2005-2006) succeeded in distributing 53,921 units of whole blood, 13,65,002 units of packed cells, 5,23,300 units of FFP, 1,55,539 units of cryo, 74,287 units of CPP, 4,78,973 units of platelet concentrates and 99,794 units of washed red blood cells across the country.

## Hospital Blood Transfusion Committees and Oversight

Since 1992, hospital blood transfusion committees have been assigned the responsibility to oversee the practice of blood transfusion in hospitals. An appropriate performance of these committees along with implementation of supervisionary, scientific and educational programs ensure a suitable procedure of blood transfusion in hospitals. There are about 326 committees in 25 provinces out of 30 in Iran. Based on the procedure approved and signed by the Iranian Minister of Health, it is not obligatory for any hospital blood transfusion committees to include any members from IBTO. The sessions of such committees are held every month or every other month according to the number of occupied hospital beds. The responsibilities of hospital blood transfusion committees are to report adverse transfusion reactions, to record the number of blood transfusion attempts and the rate of blood orders from different hospital wards, to report the causes and indications of blood transfusion, to hold and report on educational programs and to study personnel problems and any inadequacy in blood bank equipment. The committees are also responsible to provide strategies to improve blood transfusion quality in hospitals.

The study conducted by the IBTO Deputyship for Quality Control and Technical Affairs indicated that 71% of hospitals in 25 provinces of Iran have established hospital blood transfusion committees. Of these, only 43% enjoy regularly active committees and 57% are irregularly managed.

The study conducted by Hajibeigi *et al.* in Tehran hospitals indicated that of the reports prepared by the hospital blood transfusion committees, the highest pertained to adverse blood transfusion reactions (77%) and the lowest to causes and indications of blood transfusion (23%). The above study shows that these committees need to play a more active role in supervising appropriate blood use so as to lower the blood wastage rate.[3]

Two members (one physician and one technician) at each blood center across the country who undergo training courses organized by IBTO are responsible to oversee the activities of hospital blood banks and blood transfusion committees twice a year. They are not necessarily members of hospital blood transfusion committees. They monitor hospital blood banks and try to ensure appropriate use of blood and blood components. They prepare a final report incorporating details of blood storage in blood banks; blood bank equipment and commodities; cross-match tests and the methods conducted for these tests and data pertinent to blood use and other similar purposes.

## Fractionation Center

Plasma fractionation started to develop in 1976 within a small department at IBTO; it began to produce protein products prepared from human plasma and started mass production of factors VIII and IX, human albumin, normal immunoglobulin and specific anti-rabies sera. The fractionation center was inaugurated in 1994 and has an annual fractionation capacity of 80,000 liters of plasma. In 1997, it was decreed that this center should stop operating because of litigations of some hemophilia patients who claimed to be infected with HIV following the use of blood products. In those days, the intention to implement modern standards especially for new virus inactivation, heat treatment and nanofiltration methods also played a role in this regard. Since then, this center has been facing problems. However, since 2003 it has been involved in exporting the plasma collected in blood centers across Iran. In 2004, 20,000 liters of FFP and Platelet poor plasma (PPP) were exported to Octapharma, Austria and in 2005, 70,000 to Biotest AG companies, Germany; all samples were collected from non-remunerated blood donors. These two companies tested and processed the plasma components as different biologic products including gamma globulin and factors VIII and IX, and they were returned to Iran in bulk. The bulk products were divided in vials with the approval license of these companies.

## Postgraduate Education

To employ well-trained and responsible professional staff that is able to follow quality assurance and good manufacturing practices, it is necessary to have an effective academic education program. There are already several courses on hematology and blood banking held in IBTO. We also should train health practitioners to increase their awareness of standards for medical practice in prescribing blood and blood products to promote appropriate blood use thereby reducing blood loss.

So far, 37 MSc students with hematology and blood banking majors have graduated from the IBTO research center.

## New Measures

Given the regional status of IBTO and for better recognition of blood recipients and those prescribing blood transfusion, IBTO has planned special programs to improve the production of modern components and increase safety. Among these, one can refer to the establishment of apheresis units in some major blood transfusion centers to prepare blood components like platelets through apheresis. Meanwhile, due to the development of the plasma fractionation unit in Iran and greater demand for plasma as also the impossibility to ensure the needed plasma to manufacture plasma-derived pharmaceuticals through recovered plasma, there is a program to prepare plasma including normal and specific plasma like anti-rabies plasma through apheresis, which can meet the actual needs of the country next to the efforts made by the private sector. One of the most important objectives in collecting the necessary plasma to manufacture plasma-derived pharmaceuticals is to produce IVIg derived from normal plasma donated in Iran so as to improve the therapy quality of this important component.

Considering the significance of improving blood safety, the new programs of IBTO involve increasing the number of regular blood donors and encouraging first-time blood donors to change into regular donors; this is especially being followed given the studies conducted in this regard; better awareness of biography of blood donors, their motivations and their anxiety of blood donation and continuous training programs for target groups so that the current rate of 38% of regular blood donors increases to 42% by the end of 2007.

Among the future programs of IBTO, one can refer to the NAT test that has started to be implemented as a research pilot program in the Tehran Regional Educational Blood Transfusion Center based on whose results the efficiency in implementation, cost-effectiveness and experiences in its implementation are evaluated so as to be used in implementing the NAT test in other blood centers.

Since IBTO is managed nationally and decentralized, in order to improve the safety of blood and blood components prepared in IBTO and reduce the cost, there is a need for reduction of the number of blood screening tests and their centralization that is supposed to be theoretically implemented in 10 major blood centers so as to completely materialize if satisfactory.

One of the most significant problems of IBTO is the lack of full awareness of physicians and nurses regarding the appropriate use of blood and blood components, adverse blood transfusion reactions, preparation and storage criteria and transportation of different types of cellular and plasma components. Given the importance of the last cycle of blood safety, which is the appropriate use of blood and blood components, there is a need for training physicians and nurses. Thus, strategies such as inclusion of transfusion medicine credits equal to 1.5 for the two major groups of general practitioners and nurses and education of transfusion medicine specialists, whose curricula are prepared by the faculty members and experts of IBTO and forwarded to the Ministry of Health for approval. At the same time, all regional blood transfusion centers are responsible to hold a scientific seminar at least once a year regarding transfusion medicine in collaboration with the faculty members of universities of medical sciences and IBTO research center.
